# Chelerythrine-Induced Apoptotic Cell Death in HepG2 Cells Involves the Inhibition of Akt Pathway and the Activation of Oxidative Stress and Mitochondrial Apoptotic Pathway

**DOI:** 10.3390/antiox11091837

**Published:** 2022-09-18

**Authors:** Yanling Lin, Qinzhi Zhang, Baofu Xie, Haiyang Jiang, Jianzhong Shen, Shusheng Tang, Chongshan Dai

**Affiliations:** 1 College of Veterinary Medicine, China Agricultural University, Beijing 100193, China; 2 Key Biology Laboratory of Chinese Veterinary Medicine, Ministry of Agriculture and Rural Affairs, Beijing 100193, China; 3 Beijing Key Laboratory of Detection Technology for Animal-Derived Food Safety, Beijing 100193, China

**Keywords:** chelerythrine, oxidative stress, apoptosis, mitochondrial apoptotic pathway, Akt pathway

## Abstract

Chelerythrine (CHE) is a majorly harmful isoquinoline alkaloid ingredient in *Chelidonium majus* that could trigger potential hepatotoxicity, but the pivotal molecular mechanisms remain largely unknown. In the present study, CHE-induced cytotoxicity and the underlying toxic mechanisms were investigated using human HepG2 cells in vitro. Data showed that CHE treatment (at 1.25–10 μM)-induced cytotoxicity in HepG2 cells is dose-dependent. CHE treatment increased the production of ROS and induced oxidative stress in HepG2 cells. Additionally, CHE treatment triggered the loss of mitochondrial membrane potential, decreased the expression of mitochondrial complexes, upregulated the expression of Bax, CytC, and cleaved-PARP1 proteins and the activities of caspase-9 and caspase-3, and downregulated the expression of Bcl-XL, and HO-1 proteins, finally resulting in cell apoptosis. N-acetylcysteine supplementation significantly inhibited CHE-induced ROS production and apoptosis. Furthermore, CHE treatment significantly downregulated the expression of phosphorylation (p)-Akt (Ser473), p-mTOR (Ser2448), and p-AMPK (Thr172) proteins in HepG2 cells. Pharmacology inhibition of Akt promoted CHE-induced the downregulation of HO-1 protein, caspase activation, and apoptosis. In conclusion, CHE-induced cytotoxicity may involve the inhibition of Akt pathway and the activation of oxidative stress-mediated mitochondrial apoptotic pathway in HepG2 cells. This study sheds new insights into understanding the toxic mechanisms and health risks of CHE.

## 1. Introduction

Traditional Chinese medicines (TCMs) usually exhibit multiple biological functions, including antioxidant, anti-inflammatory, antimicrobial, and immune regulation activities, and play a critical role in health care [1]. To date, the therapeutic effectiveness of TCMs has been illustrated by multiple in vitro studies, animal experiments, and human clinical trials [2]. However, due to the presence of several special ingredients or toxic compounds (e.g., aristolochic acids, anthraquinones, flavonoids, and glycosides), TCMs may cause potential toxic effects to animals or humans, including neurotoxicity, nephrotoxicity, reproductive toxicity, respiratory toxicity, hepatotoxicity, and immunotoxicity; this has raised concerns worldwide [1,3,4]. Therefore, identifying the active ingredients, and understanding their toxic effects and molecular mechanisms is necessary for the safety assessment of TCMs.

*Chelidonium majus* L. is an important medical plant and has a long history of being used in the treatment of many diseases (e.g., infective diseases, cancer, chronic bronchitis, and liver disorders) in China and several European countries [5]. It has been reported that the extracts of *Chelidonium majus* L. possess a broad range of biological functions, such as anticancer, anti-inflammatory, antioxidant, antibacterial, immune regulation, and others [5,6,7,8,9,10,11]. However, recent findings show that the extract of *Chelidonium majus* L. may cause potential hepatotoxicity in humans and animals [12,13]. Chelerythrine (CHE), sanguinarine, and coptisine have been identified as the main hepatotoxic components of *Chelidonium majus* L. [14]. Notably, CHE is a benzophenanthridine alkaloid compound and has potent toxic effects via several in vivo and in vitro studies [15]. CHE is a natural inhibitor of the protein kinase C (PKC) isoenzyme, which is a critical regulator in controlling cell proliferation, differentiation, growth, and survival [16]. Pharmacological inhibition of PKC activity usually causes cell death or growth inhibition in most mammalian cells, and it is a critical factor in explaining the toxicity of CHE [16]. In addition, it was also found that CHE exposure could induce oxidative stress, mitochondrial dysfunction, cell cycle arrest, endoplasmic reticulum stress, DNA damage, and cell apoptosis in various cancer cells in vitro [17,18,19,20,21]. A recent study indicated that CHE could recognize human telomeric DNA and RNA G-quadruplexes that play critical roles in maintaining chromosome stability, finally directly causing DNA damage [22]. Recently, Zhang et al. found that CHE could trigger cell autophagy and activate autophagy flux, and this process is partly dependent on the production of reactive oxygen species (ROS) [23]. The toxic effects caused by CHE severely limited its clinical application and product development. Unfortunately, the key molecular mechanisms have so far remained unclear. Therefore, in the present study, HepG2 cells, a classic human hepatoma cell line that is used to evaluate hepatotoxicity by drugs or toxins, were used to study the cytotoxicity of CHE. Meanwhile, the underlying molecular mechanisms involved in the mitochondrial apoptotic pathway and protein kinase B (PKB, also named Akt) pathway were further analyzed. Our study will provide more insights into understanding the toxic mechanisms of CHE and potential intervention strategies against *Chelidonium majus* L.-induced toxic effects in clinical practices.

## 2. Materials and Methods

### 2.1. Chemicals and Regents

CHE (chloride form, purity ≥ 98%) was obtained from Aladdin Reagent Co., Ltd. (Shanghai, China). Sodium dodecyl sulfonate (SDS), Tris hydroxymethyl (Tris-HCl), leupeptin, aprotinin, and pepstatin A were obtained from AMRESCO Inc. (Solon, OH, USA). Phenylmethanesulfonylfluoride (PMSF), 1% (*v*/*v*) penicillin and streptomycin, LY294002, dimethyl sulfoxide (DMSO), rhodamine (Rh)123, 0.05% Trypsin-EDTA, 2′,7′-dichlorfluorescein-diacetate (DCFH-DA), N-acetylcysteine (NAC), and the Hoechst 33342 kit were all purchased from Beyotime (Haimen, China). CHE was prepared in DMSO as a 20 mM stock solution and stored at −80 °C for standby. All other regents used in the below experiments were of analysis grade.

### 2.2. Cell Cultures

Human HepG2 cells (provided by the Cell Bank of the Chinese Academy of Sciences in Shanghai, China) were employed in the present study. Cells were cultured in Dulbecco’s Modified Eagle’s Medium (DMEM) medium containing 10% fetal bovine serum (FBS) FBS (*v*/*v*) (Gibco, Grand Island, NY, USA) and 1% (*v*/*v*) penicillin-streptomycin and maintained in a cell incubator with 95% air and 5% CO_2_ at 37 °C. 

### 2.3. Measurement of Cell Viability

To assess the cytotoxicity of CHE in HepG2 cells, a Cell Counting Kit-8 (CCK-8) kit (Vazyme, Nanjing, China) was employed to test the changes in cell viability, according to the previous study [24]. Briefly, 1 × 10^4^ cells/well were seeded in a 96-well plate and cultured for 20 h; then, cells were treated with CHE at different concentrations (i.e., 1.25, 2.5, 5, and 10 μM, respectively). Cells in the vehicle control group were treated with 0.2% DMSO. After 24 h, cell viabilities were measured according to the instructions of the CCK-8 Kit. Finally, all OD values were recorded and normalized to those in the control group.

### 2.4. Measurement of Cell Apoptosis

To measure the cell apoptosis rates, an Annexin V-FITC/PI Apoptosis Detection Kit (Vazyme Biotech Co., Ltd., Nanjing, China) was employed, and the protocols were in accordance with our previous study [25]. HepG2 cells were treated with CHE at 2.5, 5, and 10 μM for 24 h, cell apoptotic rates were measured. The detailed protocols are shown in the Supplementary Materials.

To observe the nuclear morphological change, HepG2 cells were also stained with Hoechst 33342, according to our previous study [26]. In brief, HepG2 cells were treated with CHE at the various concentrations of 2.5, 5, and 10 μM for 24 h and were stained with Hoechst 33342 dye (at the final concentration of 10 μg/mL) for 30 min in the dark at room temperature. The morphological changes of cell nucleus were observed using a fluorescence microscope (Leica Microsystems, Wetzlar, Germany). Cells showing chromatin condensation or DNA fragmentation were defined as apoptotic death. 

To assess the role of the PI3K/Akt pathway in CHE-induced apoptosis, HepG2 cells were pretreated with LY294002 (at the final concentrations of 10 μM), a PI3K/Akt inhibitor, for 2 h, followed by co-treating with CHE (at the final concentrations of 10 µM) for an additional 24 h. Finally, cells were stained with Hoechst 33342 and cell apoptotic rates were counted.

### 2.5. Measurements of ATP Levels

The ATP levels in HepG2 cells were measured with the Enhanced ATP Assay Kit (Beyotime, Haimen, China), according to the commercial kit instructions. Briefly, 2 × 10^5^ cells/well were plated into a 12-well plate, then cells were exposed to the different concentrations of CHE (i.e., at 1.25, 2.5, 5, and 10 μM, respectively) for 24 h. After treatment, HepG2 cells were lysed using the 100 μL lysis buffer provided by the kit. Cells were centrifugated at 14,000× *g* for 10 min at 4 °C, then supernatants were collected and mixed with ATP detection working liquid for measurement. The values were read using a microplate luminometer (Tecan Trading AG, Männedorf, Switzerland).

### 2.6. Determination of Intracellular ROS Generation, Malondialdehyde (MDA), Catalase (CAT) and Superoxide Dismutase (SOD) 

To assess the role of oxidative stress in CHE-induced cytotoxicity, intracellular ROS production, and the biomarkers of oxidative stress, including the levels of MDA and the activities of SOD and CAT, were measured. HepG2 cells were exposed to the various doses of CHE (i.e., at 2.5, 5, and 10 μM) for 24 h. The intracellular ROS production, the levels of MDA, and the activities of SOD and CAT were measured using commercially available kits, respectively. The detailed protocol was followed by the descriptions in the previous study [27] and shown in the Suppl. Materials and Methods. 

### 2.7. Determination of Mitochondrial Membrane Potential (MMP)

Rhodamine-123 dye was employed to measure the changes in MMP, according to the published descriptions [28]. Briefly, 2 × 10^5^ cells per well were plated into a 12-well plate. After 20 h of culture, cells were exposed to various doses of CHE (i.e., at 2.5, 5, and 10 μM, respectively) for 24 h. Then, cells were washed twice with PBS and stained with Rh123 dye (at a final concentration of 1 μg/mL) for 30 min in a cell incubator. Then, cells were washed with PBS three times. Finally, a fluorescence microscope with an emission wavelength of 525 nm and an excitation wavelength of 488 nm was employed to observe the changes in MMP. For the quantitative analysis, cells were digested with 0.25% trypsin-EDTA, then cells were collected and analyzed using flow cytometry.

### 2.8. Determination of Caspase-9 and Caspase-3 Activities

The levels of caspase-9 and caspase-3 activities in HepG2 cells were examined using commercial caspase-9 and caspase-3 kits (Beyotime, Haimen, China), respectively. The detailed protocols were in line with our previous study [26]. In brief, HepG2 cells were treated with different doses of CHE (i.e., at 1.25, 2.5, 5, and 10 μM, respectively). After 24 h of treatment, cells were washed twice with PBS and lysed with the lysis buffer provided by the kits. Cell samples were then centrifuged at 14,000× *g* for 15 min, and the supernatants were collected to measure the values. The protein concentrations were examined using a BCA kit (Thermo Fisher Scientific Inc., Waltham, MA, USA). The activities of caspase-9 and caspase-3 in each sample were normalized to their protein concentrations.

### 2.9. Western Blotting

The levels of protein expression were examined using Western blotting and the detailed protocols were followed as per our previous descriptions [26]. In brief, HepG2 cells were treated with various doses of CHE (i.e., at 1.25, 2.5, 5, and 10 μM, respectively) or were treated with 0.2% DMSO as the vehicle control group. After 24 h, cells were washed twice with PBS and lysed using the RIPA lysis buffer (Beyotime, Haimen, China) with several protein inhibitors, including PMSF (at 10 μg/mL), leupeptin (at 1 μg/mL), aprotinin (at 1 μg/mL), and pepstatin A (at 1 μg/mL). All samples were lysed for 15 min and were ultrasonicated for 2 min using an Ultrasonic Processor (Branson, MO, USA) at 4 °C. Then, samples were centrifuged at 14,000× *g* at 4 °C for 15 min, and supernatants were collected. Then, the protein concentrations were measured using a BCA kit (Thermo Fisher Scientific Inc., Waltham, MA, USA). Western blotting, including protein isolation, electroporation, transfer, and antibody incubation, was performed. Several primary antibodies, including rabbit polyclonal antibodies against Bax, PARP1, HO-1, LC3, CytC, and Bcl-XL (1:1000 dilution; Proteintech, Chicago, IL, USA), phosphorylation (p)-Akt (Ser 473) (Sangon Biotech, Shanghai, China), mitochondrial oxidative phosphorylation (OXPHOS) complexes (I to V) (Abcam, Cambridge, MA, USA), p-mTOR (Ser2448), p-AMPK (Thr172) (CST, Danvers, MA, USA), and mouse monoclonal antibody against β-actin (Santa Cruz, CA, USA) were used. All antibodies were used at a 1:1000 dilution. In addition, the corresponding anti-rabbit or anti-mouse secondary antibodies (both in 1:10,000 dilution, Santa Cruz, Inc., Dallas, TX, USA) were used. Finally, the gray values of protein bands were analyzed using Image J (National Institutes of Health, Bethesda, MD, USA), and the values of protein expression were normalized to β-actin.

### 2.10. Statistical Analysis

Data in the current study are shown as mean ± standard deviation (S.D.) unless specifically mentioned. A one-way analysis of variance (ANOVA), followed by an LSD post hoc test provided by GraphPad prism 9.0 software (San Diego, CA, USA) was performed. A value of *p* less than 0.05 was considered a significant difference.

## 3. Results

### 3.1. CHE Treatment Induces the Loss of Cell Viabilities in HepG2 Cells

**Figure 1:**
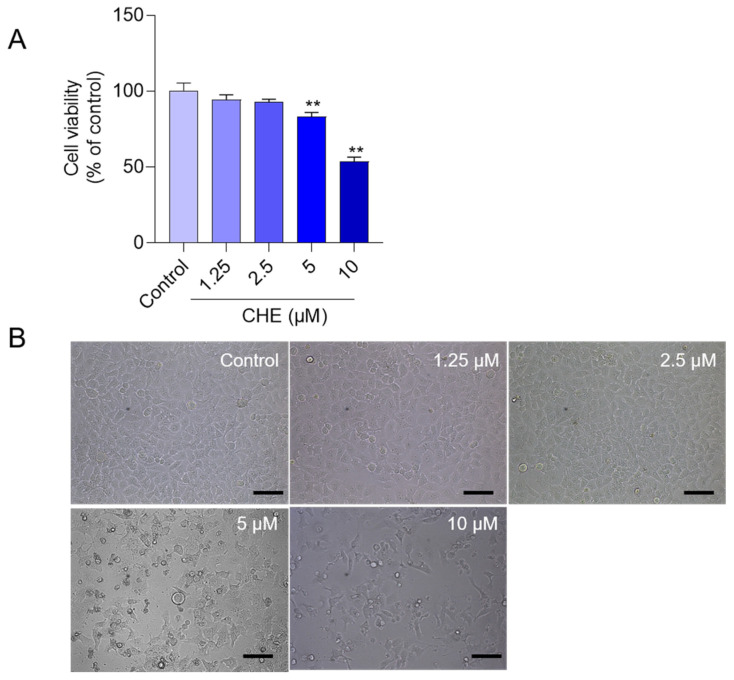
The cytotoxicity of chelerythrine (CHE) in HepG2 cells. (**A**) the effects of CHE treatment on the cell viability. HepG2 cells were treated with the different doses of CHE (at 1.25, 2.5, 5, and 10 μM) for 24 h and cell viabilities were determined using the CCK-8 method. Data were presented as mean ± S.D. from four independent experiments (*n* = 4). (**B**) the representative images of HepG2 cells treated with the different doses of CHE. Scale Bar = 50 μm. ** *p* < 0.01, compared to the control group.

CHE treatment induced a decrease in cell viabilities in HepG2 cells and was dose-dependent. As shown in Figure 1, compared to the control group, CHE exposure at the final concentrations of 1.25, 2.5, 5, and 10 μM for 24 h significantly decreased the cell viabilities of HepG2 to 94.2%, 92.9%, 83.2% (*p* < 0.01), and 53.5% (*p* < 0.01) (Figure 1A), respectively. Correspondingly, the marked decreases in cell number and abnormal morphological changes, i.e., cell body spindle-like and shrinkage changes, were detected in the 5 and 10 μM CHE treatment groups (Figure 1B). 

### 3.2. CHE Treatment Induces Cell Apoptosis

**Figure 2:**
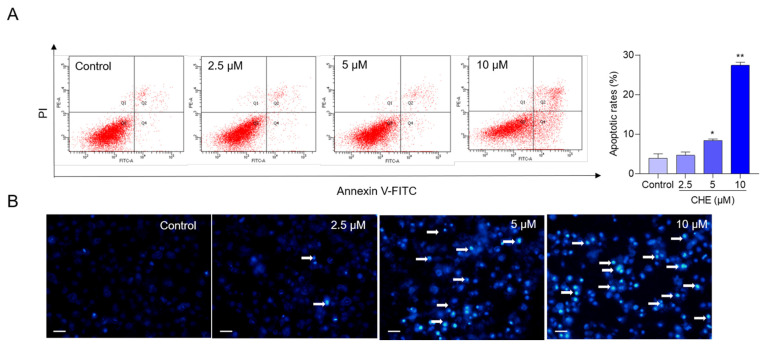
CHE treatment induces cell apoptosis in HepG2 cells. HepG2 cells were treated with various doses of CHE (i.e., at 2.5, 5, and 10 μM, respectively) for 24 h, and apoptosis rates were measured using Annexin V-FITC/PI (**A**) or Hoechst 33342 staining (**B**) methods. Data were shown as mean ± S.D. (*n* = 3). In B, the white arrows indicate the apoptotic cells. * *p* < 0.05, or ** *p* < 0.01, compared to the control group. Scale Bar = 25 μm.

CHE treatment induced apoptotic cell death of HepG2 cells, and it was in a dose-dependent manner. As shown in Figure 2A, CHE treatments at the final concentrations of 2.5, 5, and 10 μM increased the apoptotic rates to 4.7%, 8.4% (*p* < 0.05), and 27.4% (*p* < 0.01), respectively, compared to that in the vehicle control group. Consistent results were also detected using the Hoechst 33342 staining method. As shown in Figure 2B, CHE treatments at the final doses of 5 and 10 μM both increased the levels of condensed nuclei and fragmented chromatin, compared to the control group. 

### 3.3. CHE Treatment Induces Oxidative Stress

**Figure 3:**
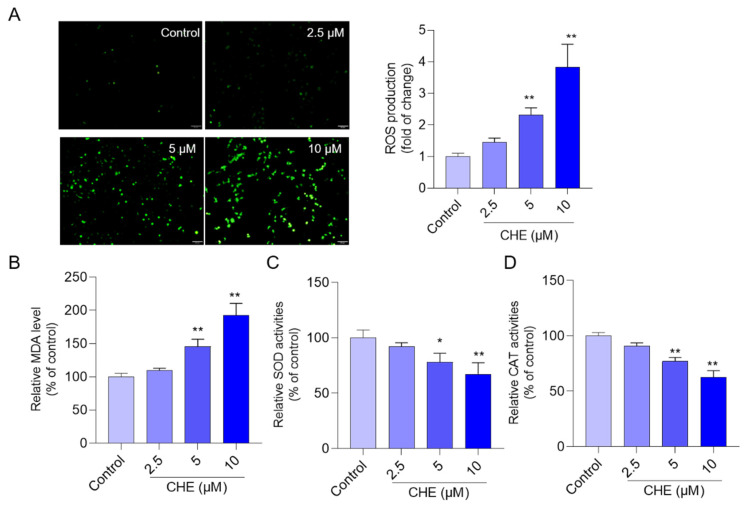
CHE treatment increases intracellular ROS production and MDA levels and decreases intracellular SOD and CAT activities in HepG2 cells. (**A**) The results of intracellular ROS production. HepG2 cells were treated with different doses of CHE (at 2.5, 5, and 10 μM, respectively) for 24 h, then cells were incubated the DMEM mediums containing 2,7-dichlorofluorescein diacetate dye. The representative images (on the left) and the fluorescence intensities (on the right) are shown. Bar = 50 μm. B-D, The levels of intracellular MDA levels, (**B**) and SOD (**C**) and CAT (**D**) activities. Data were shown as mean ± S.D. (*n* = 3). * *p* < 0.05, or ** *p* < 0.01, compared to the control group.

CHE treatment significantly induced the production of ROS, increased intracellular MDA levels, and decreased the activities of the antioxidant enzymes SOD and CAT. As shown in Figure 3, CHE treatment at the final concentrations of 2.5, 5, and 10 μM for 24 h significantly increased the levels of intracellular ROS to 1.4-, 2.3- (*p* < 0.01), and 3.8-fold (*p* < 0.01), respectively; significantly increased the intracellular MDA levels to 109.6%, 145.6% (*p* < 0.01), and 192.4% (*p* < 0.01), respectively; significantly decreased intracellular SOD activities to 92.3%, 78.1% (*p* < 0.05), and 67.3% (*p* < 0.01), respectively; and significantly decreased intracellular CAT activities to 91.1%, 76.9% (*p* < 0.05), and 62.7% (*p* < 0.01), respectively, compared to those in the control group.

### 3.4. CHE Treatment Induces Mitochondrial Dysfunction

**Figure 4:**
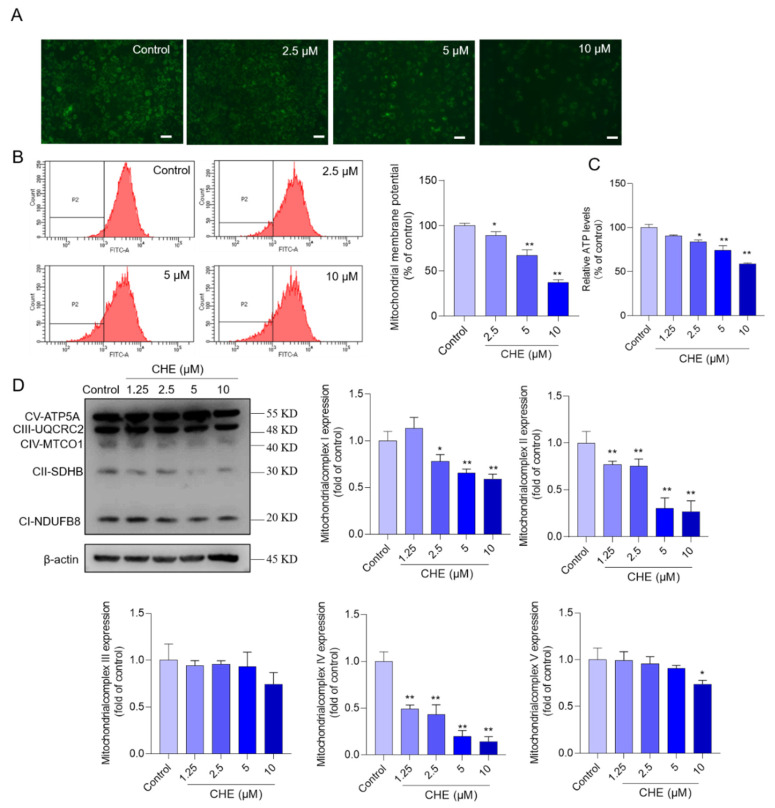
CHE treatment causes mitochondrial dysfunction in HepG2 cells. (**A**) the changes in mitochondrial membrane potential (MMP) were observed using a fluorescence microscope and the representative images are shown. Scale Bar = 50 μm. (**B**) the results of flow cytometry analysis. (**C**) the levels of ATP content. (**D**) levels of expression of mitochondrial complexes I to V subunits were measured by using western blotting (on the left). The corresponding quantitative analysis was performed (on the right). Data are shown as mean ± S.D. (*n* = 3). * *p* < 0.05, or ** *p* < 0.01, compared to that in the vehicle control group.

Fluorescence microscope observations showed that CHE treatment significantly decreased the MMP of HepG2 cells (Figure 4A). Consistently, the quantitative analysis of MMP showed that CHE treatment at the final doses of 2.5, 5, and 10 μM decreased the MMP levels to 89.4%, 66.9%, and 36.9% (all *p* < 0.05 or *p* < 0.01), respectively, compared to the control group (Figure 4B). The intracellular ATP levels were further assessed. As shown in Figure 4C, CHE exposure at the final doses of 2.5, 5, and 10 μM decreased the ATP levels to 83.9%, 74.1%, and 59.1% (all *p* < 0.05 or *p* < 0.01), respectively, compared to that in the vehicle control group. In addition, the expression of CI–V respiratory chain subunits was further examined. As shown in Figure 4D, data showed that CHE treatment at 1.25 μM significantly decreased the expression of CII and CIV, and CHE at 10 μM significantly decreased the expression of CII, CII, CIV, and CV. All CHE treatment groups did not affect the expression of CIII. 

### 3.5. CHE Treatment Activates Mitochondrial Apoptotic Pathway

**Figure 5:**
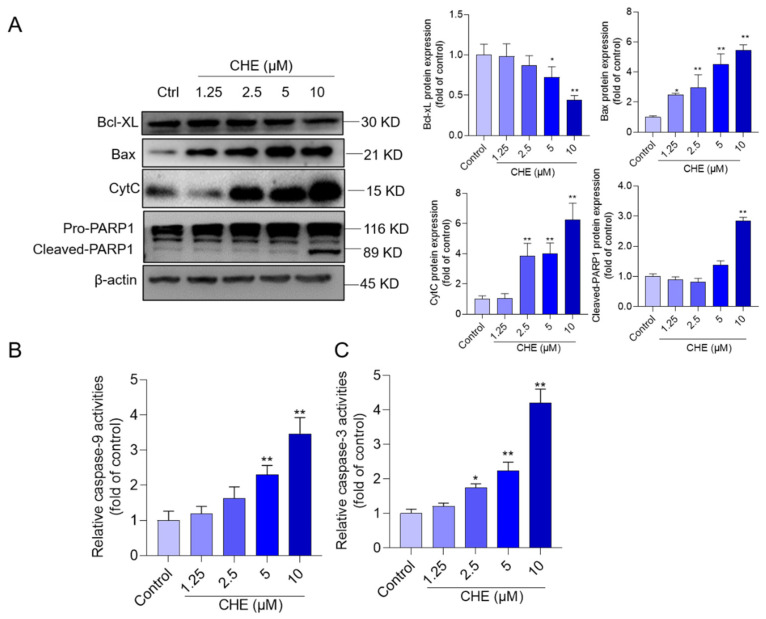
CHE treatment activates the mitochondrial apoptotic pathway in HepG2 cells. (**A**) cells were treated with various doses of CHE, i.e., 1.25, 25.5, 5, and 10 μM, respectively. After 24 h, the expressions of Bcl-XL, Bax, CytC, and PRRP1 proteins were examined using the Western blotting method. (**B**,**C**) the levels of caspase-9 (B) and caspase-3 (C) activities. Data are shown as mean ± SD (*n* = 3 independent experiments). * *p* < 0.05 or ** *p* < 0.01, compared to those in the vehicle group.

The expressions of proteins involved in the mitochondrial apoptotic pathway, including pro-apoptotic proteins Bax, CytC, and cleaved-PARP1, and the anti-apoptotic protein Bcl-XL, were examined. Compared to those in the vehicle control group, CHE treatment at the concentration range of 1.25–10 μM upregulated the expression of Bax to 2.5-, 3.0-, 4.5-, and 5.4-fold (all *p* < 0.05 or 0.01), respectively; upregulated the expression of CytC to 1.0-, 3.8- (*p* < 0.01), 4.0- (*p* < 0.01), and 6.3-fold (*p* < 0.01), respectively; and downregulated the Bcl-XL expression of 0.98-, 0.86-, 0.72- (*p* < 0.05), and 0.43-fold (*p* < 0.01), respectively. In addition, the expression of cleaved-PARP1 protein significantly increased to 2.8-fold in the 10 μM CHE treatment groups, and its expression had no significant changes in the other dose group, compared to the control group (Figure 5A). Meanwhile, significantly increased activities of caspase-9 and caspase-3 were detected. CHE treatment at the concentrations of 2.5, 5, and 10 μM upregulated caspase-9 activities to 1.6-, 2.3- (*p* < 0.01), and 3.5-fold (*p* < 0.01), and upregulated caspase-3 activities to 1.8-, 2.2-, and 4.2-fold (all *p* < 0.05 or 0.01) (Figure 5B,C), compared to those in the vehicle control group.

### 3.6. CHE Treatment Inhibits AMPK/Akt Pathway

**Figure 6:**
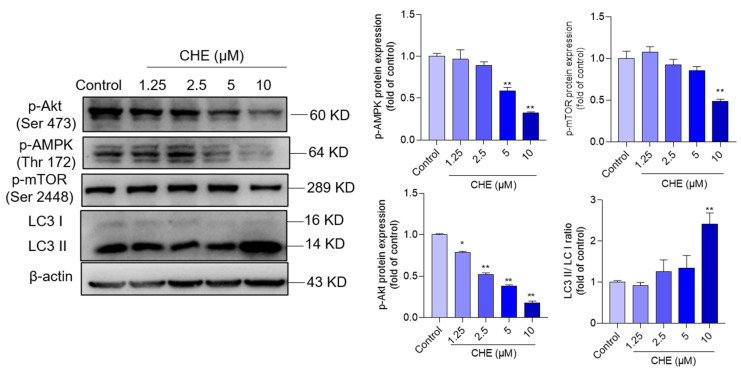
CHE treatment downregulated the expression of p-Akt, p-AMPK, and p-mTOR proteins and upregulated the ratio of LC3II/LC3I in HepG2 cells. The representative images of Western blotting are shown on the left, and the quantified analysis is shown on the right. Data were presented as mean ± S.D. (*n* = 3 independent experiments). * *p* < 0.05 or ** *p* < 0.01, compared to those in the vehicle control group.

Compared to the control group, CHE treatment at the final concentrations of 1.25–10 μM decreased the expression of p-AMPK to 0.97-, 0.89, 0.59- (*p* < 0.01), and 0.32-fold (*p* < 0.01), respectively (Figure 6), and decreased the expression of p-Akt to 0.78-, 0.52-, 0.38-, and 0.18-fold (all *p* < 0.05 or 0.01), respectively. In addition, the significant increased expression of p-mTOR and LC3II was also detected in the 10 μM CHE treatment group, compared to the untreated group (Figure 6). 

### 3.7. NAC Supplementation Improved CHE-Induced the Production of ROS and Apoptosis

The role of oxidative stress in CHE-induced cytotoxicity was further assessed. As shown in Supplementary Materials Figure S1, compared to the CHE alone treatment group, NAC supplementation significantly decreased the ROS production from 3.2-fold to 1.4-fold (*p* < 0.01). Meanwhile, NAC supplementation significantly decreased cell apoptosis rates from 43.3% to 17.6% (*p* < 0.01), compared to the CHE alone treatment group. 

### 3.8. Inhibition of Akt Pathway Promotes CHE-Induced Cytotoxicity and Apoptosis

**Figure 7:**
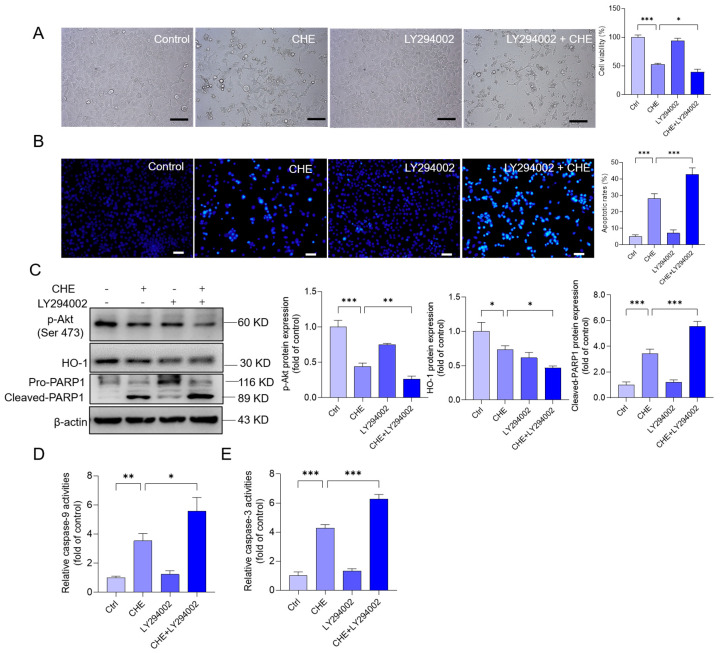
Effects of Akt inhibition on the cell viability, apoptosis, and protein expression in HepG2 cells exposed to CHE. (**A**) HepG2 cells were pretreated with LY294002 (at a final concentration of 10 μM) for 2 h, followed by treatment with CHE at 10 μM for an additional 24 h. The morphological changes (on the left) were observed and the changes in cell viability (on the right) were measured. (**B**) measurement of cell apoptosis. The representative images (on the left) and the quantitative analysis (on the right) are shown. (**C**) the expressions of p-Akt, HO-1, and PRAP1 proteins were performed by using the Western blotting method. (**D**,**E**) The levels of caspase-9 and caspase-3 activities. Data are shown as mean ± S.D. (*n* = 3 independent experiments). * *p* < 0.05, ** *p* < 0.01 or *** *p* < 0.001, indicated the significant differences between the two groups. Scale Bar = 50 μm.

As shown in Figure 7, pharmacological inhibition of Akt expression by LY294002 significantly promoted CHE-induced loss of cell viability and apoptosis. As shown in Figure 7A,B, LY294002 in cotreatment with CHE significantly decreased the cell viability from 52.6% to 39.3% (*p* < 0.05) and increased the cell apoptotic rates from 28% to 42% (*p* < 0.01), compared to the CHE alone treatment group. Moreover, LY294002 cotreatment significantly decreased the expression of p-Akt protein, then further decreased the expression of HO-1 protein and increased the expression of the cleaved PARP1 protein. In the LY294002 combination with the CHE group (i.e., LY29402 + CHE group), the expression of HO-1 protein decreased to 0.47-fold, while the expression of cleaved PARP1 increased to 5.6-fold, compared to the CHE alone treatment group (Figure 7C). Consistently, LY294002 cotreated with CHE also significantly increased the activities of caspase-9 and caspase-3 (both *p* < 0.05), compared to those in the CHE alone treatment group (Figure 7D,E). 

## 4. Discussion

As a useful medicinal plant in treating many chronic or infective diseases, *Chelidonium majus* L. has been used for more than 30 years in China and several European countries [5]. Its main active ingredients contain isoquinoline alkaloids, several flavonoids, and phenolic acids, and these compounds usually exhibit multiple biological functions, such as anti-inflammatory, antioxidant, anti-tumor, and antimicrobial properties [5,29]. Recent studies showed the consumption of *Chelidonium majus* L. may cause potential hepatotoxicity and acute hepatitis in humans, which has raised concerns worldwide [12,13,30,31]. Several isoquinoline alkaloids, including sanguinarine, CHE, chelidonine, coptisine, and berberine, were detected in the extract of *Chelidonium majus* L. [5]. Among these several alkaloids, sanguinarine, CHE, and coptisine were more toxic than the others, and these three compounds were also considered potential toxic compounds [14]. Ulrichova and colleagues showed that a signal dose of sanguinarine or CHE (i.e., 10 mg/kg/day) could cause acute necrosis in the liver tissues of rats [32]. It was also found that intravenous injection of CHE at a dose of 5 mg/kg could induce the production of ROS and apoptosis in the cardiac myocytes of rats [18]. CHE has been considered as one of the most powerful toxin compounds in the extract of *Chelidonium majus* L. Therefore, deeply understanding the precise toxic mechanisms of CHE is required for the clinical application of *Chelidonium majus* L. in humans and animals. 

In this study, data showed that CHE treatment in the dose range of 1.25–10 μM exhibited marked cytotoxicity and it is dose-dependent (Figure 1). Similarly, several previous studies showed that CHE at the dose ranges of 2.5–20 μM exhibited potent cytotoxicity in various cell lines, including HL-7702 cells (i.e., a liver cell line derived from normal human liver cells, also called L02 cells), human renal cancer cell lines (i.e., Caki and 786-O), HeLa, murine embryonic fibroblasts, H1299 cells, and A549 cells (i.e., a human epithelial cell line derived from lung carcinoma tissue) [14,20,23,33]. Furthermore, our current study found CHE could induce oxidative stress, mitochondrial dysfunction, and cell apoptosis in HepG2 cells (Figure 2, Figure 3, Figure 4 and Figure 5). Mechanistically, our data from Figure 6 and Figure 7 showed CHE-induced cytotoxicity and apoptosis may be partly attributed to the activation of the mitochondrial apoptotic pathway and the inhibition of the Akt pathway. 

Oxidative stress could be induced by the imbalance between the production of intracellular ROS and antioxidant systems and it plays a critical role in the process of cytotoxicity caused by various exogenous compounds, such as mycotoxins (e.g., T-2 toxin), heavy metals (e.g., copper), or drugs (e.g., quinocetone, colistin, and furazolidone) [26,27,34,35,36]. It is well known that intracellular ROS contains the superoxide anion (O_2_^•−^), hydroxyl radicals (OH·), and hydrogen peroxide (H_2_O_2_) [37]. Corresponding, intracellular antioxidant enzyme SOD could catalyze the dismutation of O_2_^•−^ into oxygen and H_2_O_2_, and the H_2_O_2_ molecule was further decomposed into water and oxygen, which had no toxic effects to cells [37]. In this study, our data found that CHE treatment significantly increased intracellular ROS production in a dose-dependent manner (Figure 3). In the 5 μM and 10 μM CHE treatment groups, the activities of SOD and CAT were also significantly downregulated (Figure 3). Consistently, CHE treatment also significantly increased the levels of MDA (Figure 3), a biomarker of oxidative stress and an indicator of the peroxidation of membrane lipids [37]. Similarly, Xie et al. showed that CHE treatment at >3 μM could induce a significant increase in ROS in H1975 cells [38]. The previous study also found that CHE-induced oxidative stress and cell cytotoxicity are partly dependent on its effect on glutathione (GSH) depletion, which was therefore partly reversed by NAC (i.e., a precursor to GSH) supplementation [19]. Indeed, in the present study, NAC supplementation could also significantly reduce CHE-induced cell death (Supplementary Materials Figure S1). Taken together, our results indicated that CHE could trigger the production of ROS and induce oxidative stress in HepG2 cells. 

Excessive ROS production in mammalian cells could directly damage DNA, proteins, lipids, and other biomolecules, resulting in cell death, which finally results in toxic damage in tissues or organs of humans or animals [39]. Kemény-Beke et al. demonstrated that CHE at the lower concentration (i.e., 1 μg/mL for 24 h) could induce marked apoptosis in human OCM-1 cells, but not necrosis [40]. Consistently, CHE-induced apoptotic cell death could be reversed by NAC supplementation, a special ROS inhibitor [20]. In the present study, marked apoptotic cell death and DNA damage in CHE-treated HepG2 cells (i.e., CHE treatment at the doses of 5 or 10 μM for 24 h) were detected by Annexin V-FITC/PI Hoechst 33342 methods (Figure 2), respectively. Our results also showed that inhibition of oxidative stress by NAC supplementation significantly inhibits CHE-induced apoptosis (Supplementary Materials Figure S1). This evidence indicates CHE-induced cytotoxicity and apoptosis is partly dependent on the production of ROS. 

Mitochondria are the major producers of ROS and they are also the main target of ROS-induced signaling [41]. Mitochondria are also critical “players” in apoptosis [37]. Several studies have shown that CHE exposure could induce mitochondrial dysfunction, which is charactered by decreased MMP, the formation of membrane permeability, and the suppression of the mitochondrial respiratory chain in cells [19,33,42]. An earlier study reported that low concentrations of CHE could inhibit energy transfer and coupled respiration in rat liver mitochondria [43]. Similarly, Yang et al.’s study found that CHE could induce apoptosis may involve the dysfunction of mitochondrial ETC and did not involve the production of ROS [44]. In the study, data found that CHE treatment significantly decreased the protein expression of mitochondrial CI, CII, CIV, and CV (Figure 4). Mitochondrial complexes are also called oxidative phosphorylation complexes, which play a critical role in maintaining the activities of the mitochondrial electron transport chain (ETC) and the production of ATP [45]. Not surprisingly, significantly decreased ATP levels were detected in CHE-treated HepG2 cells (Figure 4). Notably, significant decreases in the levels of mitochondrial CII and IV in the 1.25 μM CHE treatment group, more sensitive than other mitochondrial complexes (Figure 4). The previous study showed that mitochondrial CII-deficiency could cause a significant increase in mitochondrial ROS [46,47]. Taken together, these data indicated that CHE-induced mitochondrial dysfunction involved the inhibition of mitochondrial ETC and ATP production. Mitochondrial complexes II and IV may be the important targets of CHE-induced cytotoxicity. The precise molecular mechanisms involved in the crosstalk between mitochondrial ETC and apoptosis still need further investigation.

The imbalance of the expressions between anti-apoptotic and pro-apoptotic proteins (such as the increase of Bax/Bcl-XL ratio) could increase the formation of mitochondrial permeability transition pore and trigger the releases of CytC from mitochondria into the cytosol, cascading to induce the activation of caspase-9 and caspase-3, finally resulting in apoptotic cell death [48]. The activation of caspase-3 could also induce the cleavage of PARP1 protein, a critical factor in regulating cell death or cell survival after DNA damage [49]. A previous study found that CHE treatment significantly upregulated the expression of pro-apoptotic proteins of Bax, Bad, and Bak proteins and downregulated the expression of anti-apoptotic proteins of Bcl-2, Bcl-XL, and Mcl-1, resulting in cell apoptosis in HeLa cells [33,50]. CHE is also considered a natural inhibitor of Bcl-XL [51]. The Zhang et al. study also found CHE exposure could induce cell apoptosis in SMMC-7721 cells by activating the mitochondrial apoptosis pathway [52]. In line with these findings, our results showed that CHE treatment significantly induced the loss of MMP, upregulated the expression of Bax, CytC, and cleavage of cleaved PARP1, and downregulated the expression of Bcl-2 protein (Figure 5). Taken together, the data from our current study indicated CHE-induced apoptotic cell death in HepG2 cells involves the activation of the mitochondrial apoptosis pathway. 

CHE treatment could upregulate the expression of autophagy proteins and activate autophagy flux, and it was partly dependent on the production of ROS [19]. mTORC1 is a central regulator of autophagy [53]. Akt is an important regulator of mTOR, which is usually considered to be a critical autophagy suppressor [53]. In addition, Akt signaling also plays a critical role in the processes of cell growth, proliferation, senescence, and death [54]. It has been demonstrated that the inhibition of the Akt/mTOR pathway plays an important role in several drugs (i.e., colistin, ivermectin, and furazolidone) or toxins (e.g., T-2 toxin, arsenic, and cadmium)-induced cytotoxicity and autophagy [26,55,56,57,58]. A previous study reported that CHE treatment at 2 μM for 2 h could significantly abolish the expression of p-Akt protein [19]. In the present study, our results showed that CHE treatment significantly downregulated the expression of p-Akt and p-mTOR (Figure 6). CHE also significantly upregulated the expression of LC3II protein, a protein marker of autophagy (Figure 6). Furthermore, pharmacological inhibition of Akt by LY294002 significantly increased CHE-induced expression of cleaved PARP-1 and apoptotic rates (Figure 7). Meanwhile, inhibition of Akt exacerbated the inhibition of HO-1 protein and increased the levels of caspase-9 and caspase-3 activities caused by CHE (Figure 7). In addition, our results showed that the changes of Akt were earlier than mTOR, indicating the other functions of Akt that are not dependent on mTOR were activated (Figure 6). It has been demonstrated that the activation of Akt could upregulate the expression of HO-1 via activating the transcriptional activity of Nrf2 [59], and the induction of HO-1 protein could effectively protect against CHE-induced cytotoxicity, oxidative stress, and apoptosis [19]. Therefore, these data indicate that the inhibition of Akt signal-mediated loss of cell viability may involve the mTOR-dependent and independent pathway. 

In addition, our present study also found that CHE treatment significantly downregulated the expression of the serine/threonine kinase AMP-activated protein kinase (AMPK) protein (i.e., phosphorylation at Thr172) (Figure 6). Under low energy stress, AMPK expression could be upregulated, and it could phosphorylate several specific enzymes and growth control nodes to increase ATP generation through promoting OXPHOS or decrease ATP consumption [60]. Therefore, the inhibition of AMPK by CHE treatment may exacerbate CHE-induced ATP deficiency in HepG2 cells. Very recently, Cai et al. found that CHE treatment upregulated the expression of p-AMPK protein and downregulated the expression of p-mTOR, finally inducing autophagy in HFLS-RA cells [61]. Taken together, these data indicate the regulatory effects of CHE in the different cell lines may be different. To address the precise molecular mechanisms of CHE, more investigations are required in the future. 

In the present study, we just studied the toxic effects and underlying molecular mechanisms of CHE. This is the limitation of this present study. Although CHE is the most toxic compound in the extract of *Chelidonium majus*, it cannot completely mimic its toxic effect. Because other compounds in the extract of *Chelidonium majus* may provide a protective role or offer a synergistic toxic effect. The toxicity of the extract of *Chelidonium majus* is more complex than any single ingredient. Therefore, the integrative and contrastive combinations of monomeric components and extracts of *Chelidonium majus* are required in the next studies. 

## 5. Conclusions

**Figure 8:**
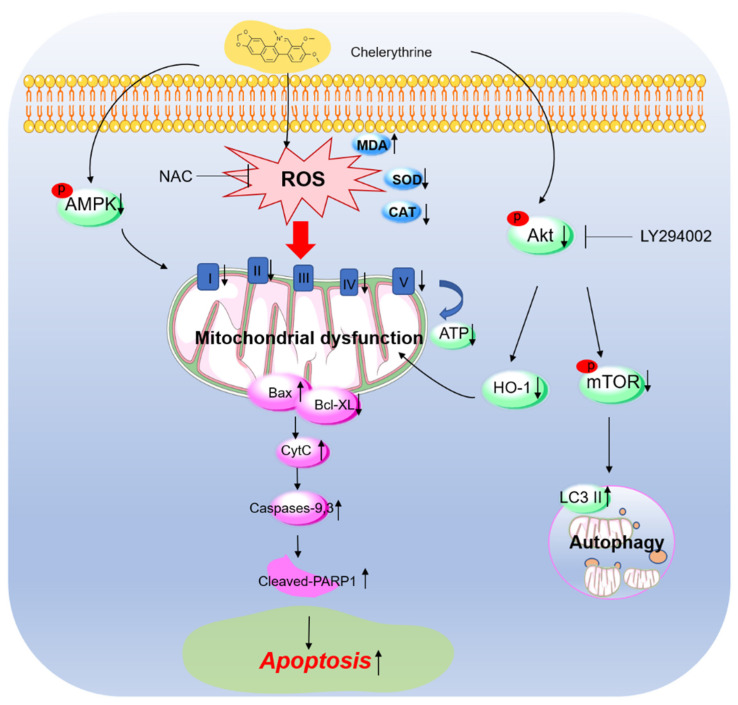
A proposed mechanism of CHE-induced apoptosis in HepG2 cells. CHE exposure induces the production of excessive ROS with a decrease in the levels of antioxidant enzymes SOD and CAT activities, finally resulting in oxidative stress. CHE exposure also disturbed the mitochondrial respiratory chain activities and decreased the expression of complexes I, II, IV, and V and intracellular ATP production, finally triggering mitochondrial dysfunction and the mitochondrial apoptotic pathway. This process may be further exacerbated by the inhibition of AMPK by CHE. Additionally, CHE treatment could downregulate the Akt pathway, which then promoted CHE-induced oxidative stress, caspase activation, and apoptosis via the inhibition of HO-1 expression or triggered autophagy via the mTOR pathway.

Our current study reveals that CHE exposure could induce excessive ROS production and trigger oxidative stress and mitochondrial apoptotic pathways, finally resulting in apoptotic cell death in HepG2 cells (Figure 8). CHE exposure could also inhibit the expression of mitochondrial complex proteins and the production of ATP, resulting in mitochondrial dysfunction, which may be further exacerbated by CHE-caused inhibition of AMPK (Figure 8). Additionally, CHE treatment could downregulate the Akt pathway, then promote CHE-induced oxidative stress, caspase activation, and apoptosis (Figure 8). Our current study sheds new insights into understanding the toxic mechanisms and health risks of CHE and *Chelidonium majus*-related products. 

## Data Availability

Data is contained within the article or supplementary material.

## Supplementary Materials

The following supporting information can be downloaded at: https://www.mdpi.com/article/10.3390/antiox11091837/s1, Figure S1: NAC supplementation attenuates CHE-induced the production of ROS and apoptosis in HepG2 cells.

## References

[1] Cai P., Qiu H., Qi F., Zhang X. (2019). The toxicity and safety of traditional Chinese medicines: Please treat with rationality. Biosci. Trends.

[2] Yip T.C., Lee H.W., Chan W.K., Wong G.L., Wong V.W. (2022). Asian perspective on NAFLD-associated HCC. J. Hepatol..

[3] Yang B., Xie Y., Guo M., Rosner M.H., Yang H., Ronco C. (2018). Nephrotoxicity and Chinese Herbal Medicine. Clin. J. Am. Soc. Nephrol..

[4] Brown A.C. (2017). Kidney toxicity related to herbs and dietary supplements: Online table of case reports. Part 3 of 5 series. Food Chem. Toxicol..

[5] Colombo M.L., Bosisio E. (1996). Pharmacological activities of *Chelidonium majus* L. (Papaveraceae). Pharmacol. Res..

[6] Zielińska S., Wójciak-Kosior M., Dziągwa-Becker M., Gleńsk M., Sowa I., Fijałkowski K., Rurańska-Smutnicka D., Matkowski A., Junka A. (2019). The Activity of Isoquinoline Alkaloids and Extracts from *Chelidonium majus* against Pathogenic Bacteria and *Candida* sp.. Toxins.

[7] Nile S.H., Wang H., Nile A., Lin X., Dong H., Venkidasamy B., Sieniawska E., Enkhtaivan G., Kai G. (2021). Comparative analysis of metabolic variations, antioxidant potential and cytotoxic effects in different parts of *Chelidonium majus* L.. Food Chem. Toxicol..

[8] Gardin N.E., Braga A.J. (2021). Greater celandine (*Chelidonium majus* L.) for COVID-19: A twenty-case series. Phytother. Res..

[9] Capistrano I.R., Wouters A., Lardon F., Gravekamp C., Apers S., Pieters L. (2015). In vitro and in vivo investigations on the antitumour activity of Chelidonium majus. Phytomedicine Int. J. Phytother. Phytopharm..

[10] Zielińska S., Czerwińska M.E., Dziągwa-Becker M., Dryś A., Kucharski M., Jezierska-Domaradzka A., Płachno B.J., Matkowski A. (2020). Modulatory Effect of Chelidonium majus Extract and Its Alkaloids on LPS-Stimulated Cytokine Secretion in Human Neutrophils. Molecules.

[11] Warowicka A., Popenda Ł., Bartkowiak G., Musidlak O., Litowczenko-Cybulska J., Kuźma D., Nawrot R., Jurga S., Goździcka-Józefiak A. (2019). Protoberberine compounds extracted from *Chelidonium majus* L. as novel natural photosensitizers for cancer therapy. Phytomedicine Int. J. Phytother. Phytopharm..

[12] Pantano F., Mannocchi G., Marinelli E., Gentili S., Graziano S., Busardò F.P., di Luca N.M. (2017). Hepatotoxicity induced by greater celandine (*Chelidonium majus* L.): A review of the literature. Eur. Rev. Med. Pharmacol. Sci..

[13] Teschke R., Glass X., Schulze J. (2011). Herbal hepatotoxicity by Greater Celandine (*Chelidonium majus*): Causality assessment of 22 spontaneous reports. Regul. Toxicol. Pharmacol..

[14] Wu C., Wang X., Xu M., Liu Y., Di X. (2019). Intracellular Accumulation as an Indicator of Cytotoxicity to Screen Hepatotoxic Components of *Chelidonium majus* L. by LC-MS/MS. Molecules.

[15] Shen Y., Zhu C., Wang Y., Xu J., Xue R., Ji F., Wu Y., Wu Z., Zhang W., Zheng Z. (2020). Evaluation the binding of chelerythrine, a potentially harmful toxin, with bovine serum albumin. Food Chem. Toxicol..

[16] Chmura S.J., Dolan M.E., Cha A., Mauceri H.J., Kufe D.W., Weichselbaum R.R. (2000). In vitro and in vivo activity of protein kinase C inhibitor chelerythrine chloride induces tumor cell toxicity and growth delay in vivo. Clin. Cancer Res. Off. J. Am. Assoc. Cancer Res..

[17] Wan K.F., Chan S.L., Sukumaran S.K., Lee M.C., Yu V.C. (2008). Chelerythrine induces apoptosis through a Bax/Bak-independent mitochondrial mechanism. J. Biol. Chem..

[18] Yamamoto S., Seta K., Morisco C., Vatner S.F., Sadoshima J. (2001). Chelerythrine rapidly induces apoptosis through generation of reactive oxygen species in cardiac myocytes. J. Mol. Cell. Cardiol..

[19] Medvetz D., Sun Y., Li C., Khabibullin D., Balan M., Parkhitko A., Priolo C., Asara J.M., Pal S., Yu J. (2015). High-throughput drug screen identifies chelerythrine as a selective inducer of death in a TSC2-null setting. Mol. Cancer Res..

[20] He H., Zhuo R., Dai J., Wang X., Huang X., Wang H., Xu D. (2020). Chelerythrine induces apoptosis via ROS-mediated endoplasmic reticulum stress and STAT3 pathways in human renal cell carcinoma. J. Cell. Mol. Med..

[21] Vrba J., Dolezel P., Vicar J., Modrianský M., Ulrichová J. (2008). Chelerythrine and dihydrochelerythrine induce G1 phase arrest and bimodal cell death in human leukemia HL-60 cells. Toxicol. Vitr. Int. J. Publ. Assoc. BIBRA.

[22] Bai L.P., Hagihara M., Nakatani K., Jiang Z.H. (2014). Recognition of chelerythrine to human telomeric DNA and RNA G-quadruplexes. Sci. Rep..

[23] Tang Z.H., Cao W.X., Wang Z.Y., Lu J.H., Liu B., Chen X., Lu J.J. (2017). Induction of reactive oxygen species-stimulated distinctive autophagy by chelerythrine in non-small cell lung cancer cells. Redox Biol..

[24] Dai C., Li H., Wang Y., Tang S., Velkov T., Shen J. (2021). Inhibition of Oxidative Stress and ALOX12 and NF-κB Pathways Contribute to the Protective Effect of Baicalein on Carbon Tetrachloride-Induced Acute Liver Injury. Antioxidants.

[25] Dai C., Li M., Sun T., Zhang Y., Wang Y., Shen Z., Velkov T., Tang S., Shen J. (2022). Colistin-induced pulmonary toxicity involves the activation of NOX4/TGF-β/mtROS pathway and the inhibition of Akt/mTOR pathway. Food Chem. Toxicol..

[26] Zhang Y., Sun T., Li M., Lin Y., Liu Y., Tang S., Dai C. (2022). Ivermectin-Induced Apoptotic Cell Death in Human SH-SY5Y Cells Involves the Activation of Oxidative Stress and Mitochondrial Pathway and Akt/mTOR-Pathway-Mediated Autophagy. Antioxidants.

[27] Dai C., Ciccotosto G.D., Cappai R., Tang S., Li D., Xie S., Xiao X., Velkov T. (2018). Curcumin Attenuates Colistin-Induced Neurotoxicity in N2a Cells via Anti-inflammatory Activity, Suppression of Oxidative Stress, and Apoptosis. Mol. Neurobiol..

[28] Dai C., Li B., Zhou Y., Li D., Zhang S., Li H., Xiao X., Tang S. (2016). Curcumin attenuates quinocetone induced apoptosis and inflammation via the opposite modulation of Nrf2/HO-1 and NF-kB pathway in human hepatocyte L02 cells. Food Chem. Toxicol..

[29] Liao W., He X., Yi Z., Xiang W., Ding Y. (2018). Chelidonine suppresses LPS-Induced production of inflammatory mediators through the inhibitory of the TLR4/NF-κB signaling pathway in RAW264.7 macrophages. Biomed. Pharmacother..

[30] Moro P.A., Cassetti F., Giugliano G., Falce M.T., Mazzanti G., Menniti-Ippolito F., Raschetti R., Santuccio C. (2009). Hepatitis from Greater celandine (*Chelidonium majus* L.): Review of literature and report of a new case. J. Ethnopharmacol..

[31] Teschke R., Frenzel C., Glass X., Schulze J., Eickhoff A. (2012). Greater Celandine hepatotoxicity: A clinical review. Ann. Hepatol..

[32] Ulrichová J., Walterová D., Vavrecková C., Kamarád V., Símánek V.m. (1996). Cytotoxicity of Benzo[c]phenanthridinium Alkaloids in Isolated Rat Hepatocytes. Phytother. Res..

[33] Yang T., Xu R., Su Q., Wang H., Liu F., Dai B., Wang B., Zhang Y. (2020). Chelerythrine hydrochloride inhibits proliferation and induces mitochondrial apoptosis in cervical cancer cells via PI3K/BAD signaling pathway. Toxicol. Vitr. Int. J. Publ. Assoc. BIBRA.

[34] Sun T., Zhang Q., Li M., Tang S., Dai C. (2022). T-2 Toxin Induces Apoptotic Cell Death and Protective Autophagy in Mouse Microglia BV2 Cells. J. Fungi.

[35] Deng S., Tang S., Dai C., Zhou Y., Yang X., Li D., Xiao X. (2016). P21(Waf1/Cip1) plays a critical role in furazolidone-induced apoptosis in HepG2 cells through influencing the caspase-3 activation and ROS generation. Food Chem. Toxicol..

[36] Dai C., Liu Q., Li D., Sharma G., Xiong J., Xiao X. (2020). Molecular Insights of Copper Sulfate Exposure-Induced Nephrotoxicity: Involvement of Oxidative and Endoplasmic Reticulum Stress Pathways. Biomolecules.

[37] Dai C., Li D., Gong L., Xiao X., Tang S. (2016). Curcumin Ameliorates Furazolidone-Induced DNA Damage and Apoptosis in Human Hepatocyte L02 Cells by Inhibiting ROS Production and Mitochondrial Pathway. Molecules.

[38] Xie Y.J., Gao W.N., Wu Q.B., Yao X.J., Jiang Z.B., Wang Y.W., Wang W.J., Li W., Hussain S., Liu L. (2020). Chelidonine selectively inhibits the growth of gefitinib-resistant non-small cell lung cancer cells through the EGFR-AMPK pathway. Pharmacol. Res..

[39] Dai C., Tang S., Li D., Zhao K., Xiao X. (2015). Curcumin attenuates quinocetone-induced oxidative stress and genotoxicity in human hepatocyte L02 cells. Toxicol. Mech. Methods.

[40] Kemény-Beke A., Aradi J., Damjanovich J., Beck Z., Facskó A., Berta A., Bodnár A. (2006). Apoptotic response of uveal melanoma cells upon treatment with chelidonine, sanguinarine and chelerythrine. Cancer Lett..

[41] Kornfeld O.S., Hwang S., Disatnik M.H., Chen C.H., Qvit N., Mochly-Rosen D. (2015). Mitochondrial reactive oxygen species at the heart of the matter: New therapeutic approaches for cardiovascular diseases. Circ. Res..

[42] Kumar S., Acharya A. (2014). Chelerythrine induces reactive oxygen species-dependent mitochondrial apoptotic pathway in a murine T cell lymphoma. Tumour Biol. J. Int. Soc. Oncodevelopmental Biol. Med..

[43] Vallejos R.H., Rizzotto M.G. (1972). Effect of chelerythrine on mitochondrial energy coupling. FEBS Lett..

[44] Funakoshi T., Aki T., Nakayama H., Watanuki Y., Imori S., Uemura K. (2011). Reactive oxygen species-independent rapid initiation of mitochondrial apoptotic pathway by chelerythrine. Toxicol. Vitr. Int. J. Publ. Assoc. BIBRA.

[45] Chaban Y., Boekema E.J., Dudkina N.V. (2014). Structures of mitochondrial oxidative phosphorylation supercomplexes and mechanisms for their stabilisation. Biochim. Biophys. Acta.

[46] Mbaya E., Oulès B., Caspersen C., Tacine R., Massinet H., Pennuto M., Chrétien D., Munnich A., Rötig A., Rizzuto R. (2010). Calcium signalling-dependent mitochondrial dysfunction and bioenergetics regulation in respiratory chain Complex II deficiency. Cell Death Differ..

[47] Kozieł R., Pircher H., Kratochwil M., Lener B., Hermann M., Dencher N.A., Jansen-Dürr P. (2013). Mitochondrial respiratory chain complex I is inactivated by NADPH oxidase Nox4. Biochem. J..

[48] Chen Q., Xu H., Xu A., Ross T., Bowler E., Hu Y., Lesnefsky E.J. (2015). Inhibition of Bcl-2 sensitizes mitochondrial permeability transition pore (MPTP) opening in ischemia-damaged mitochondria. PLoS ONE.

[49] Hong S.J., Dawson T.M., Dawson V.L. (2004). Nuclear and mitochondrial conversations in cell death: PARP-1 and AIF signaling. Trends Pharmacol. Sci..

[50] Gahl R.F., Dwivedi P., Tjandra N. (2016). Bcl-2 proteins bid and bax form a network to permeabilize the mitochondria at the onset of apoptosis. Cell Death Dis..

[51] Chan S.L., Lee M.C., Tan K.O., Yang L.K., Lee A.S., Flotow H., Fu N.Y., Butler M.S., Soejarto D.D., Buss A.D. (2003). Identification of chelerythrine as an inhibitor of BclXL function. J. Biol. Chem..

[52] Zhang Z.F., Guo Y., Zhang J.B., Wei X.H. (2011). Induction of apoptosis by chelerythrine chloride through mitochondrial pathway and Bcl-2 family proteins in human hepatoma SMMC-7721 cell. Arch. Pharmacal Res..

[53] Kim Y.C., Guan K.L. (2015). mTOR: A pharmacologic target for autophagy regulation. J. Clin. Investig..

[54] Hemmings B.A., Restuccia D.F. (2012). PI3K-PKB/Akt pathway. Cold Spring Harb. Perspect. Biol..

[55] Pi H., Li M., Zou L., Yang M., Deng P., Fan T., Liu M., Tian L., Tu M., Xie J. (2019). AKT inhibition-mediated dephosphorylation of TFE3 promotes overactive autophagy independent of MTORC1 in cadmium-exposed bone mesenchymal stem cells. Autophagy.

[56] Wang J., Yang C., Yuan Z., Yi J., Wu J. (2018). T-2 Toxin Exposure Induces Apoptosis in TM3 Cells by Inhibiting Mammalian Target of Rapamycin/Serine/Threonine Protein Kinase(mTORC2/AKT) to Promote Ca(2+)Production. Int. J. Mol. Sci..

[57] Dai C., Ciccotosto G.D., Cappai R., Wang Y., Tang S., Hoyer D., Schneider E.K., Velkov T., Xiao X. (2018). Rapamycin Confers Neuroprotection against Colistin-Induced Oxidative Stress, Mitochondria Dysfunction, and Apoptosis through the Activation of Autophagy and mTOR/Akt/CREB Signaling Pathways. ACS Chem. Neurosci..

[58] Deng S., Tang S., Zhang S., Zhang C., Wang C., Zhou Y., Dai C., Xiao X. (2015). Furazolidone induces apoptosis through activating reactive oxygen species-dependent mitochondrial signaling pathway and suppressing PI3K/Akt signaling pathway in HepG2 cells. Food Chem. Toxicol..

[59] Hao Y., Li Y., Liu J., Wang Z., Gao B., Zhang Y., Wang J. (2021). Protective Effect of Chrysanthemum morifolium cv. Fubaiju Hot-Water Extracts Against ARPE-19 Cell Oxidative Damage by Activating PI3K/Akt-Mediated Nrf2/HO-1 Signaling Pathway. Front. Nutr..

[60] Herzig S., Shaw R.J. (2018). AMPK: Guardian of metabolism and mitochondrial homeostasis. Nat. Rev. Mol. Cell Biol..

[61] Cai J., Zhang L.C., Zhao R.J., Pu L.M., Chen K.Y., Nasim A.A., Leung E.L., Fan X.X. (2022). Chelerythrine ameliorates rheumatoid arthritis by modulating the AMPK/mTOR/ULK-1 signaling pathway. Phytomedicine Int. J. Phytother. Phytopharm..

