# Supplementary material for: Chelerythrine-Induced Apoptotic Cell Death in HepG2 Cells Involves the Inhibition of Akt Pathway and the Activation of Oxidative Stress and Mitochondrial Apoptotic Pathway
Source: Antioxidants (Basel). 2022 Sep 18;11(9):1837. doi: 10.3390/antiox11091837 (PMC9495744; doi:10.3390/antiox11091837)
Supplement: Supplementary file 1 [file antioxidants-11-01837-s001.zip › antioxidants-1904106-supplementary.pdf]

Suppl. Materials and Methods.

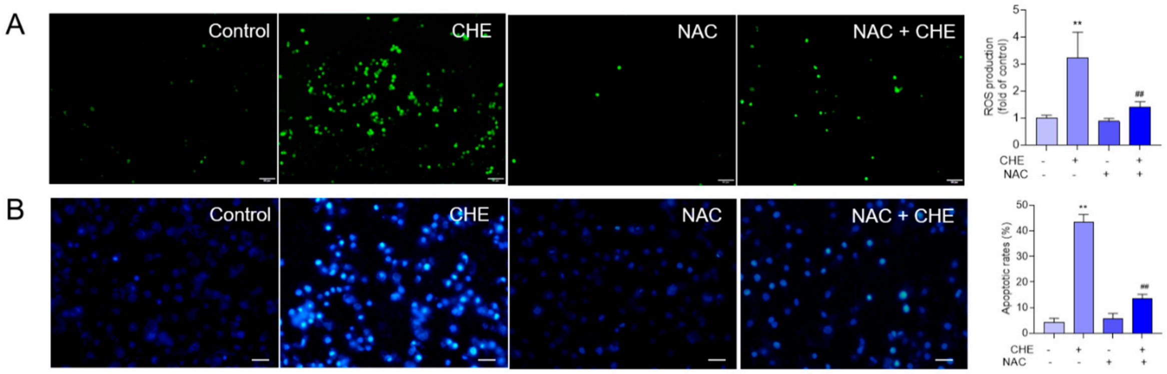

Suppl. Figure S1 NAC supplementation attenuates CHE-induced the production of ROS and apoptosis in HepG2 cells. A, the levels of ROS production. B, Measurement of cell apoptosis. The representative images were obtained using a fluorescence microscope. All results were presented as mean ± SD (n =3 independent experiments). *P<0.05 or **P<0.01, compared to the control group. Scale Bar=25 μm.
